# The microbial metabolite I3A inhibits ferroptosis and the effectiveness of redox-based cancer therapy

**DOI:** 10.1016/j.jbc.2025.111004

**Published:** 2025-12-05

**Authors:** Zixuan Guo, Lei Cui, Ruhui Yao, Yanxun Lin, Zining Wang, Huan Jin, Hui Guo, Chunyuan Xie, Lin Li, Peng Huang, Xiaojun Xia

**Affiliations:** 1 State Key Laboratory of Oncology in South China, Guangdong Provincial Clinical Research Center for Cancer, Sun Yat-Sen University Cancer Center, Guangzhou, China; 2 Department of Medical Oncology, The Sixth Affiliated Hospital, Sun Yat-Sen University, Guangzhou, China; 3 Jiangxi Provincial Key Laboratory for Precision Pathology and Intelligent Diagnosis, Department of Pathology and Institute of Molecular Pathology, The First Affiliated Hospital, Jiangxi Medical College, Nanchang University, Nanchang, China; 4 Metabolic Innovation Center, Zhongshan School of Medicine, Sun Yat-Sen University, Guangzhou, China; 5 Hainan Academy of Medical Sciences, Hainan Medical University, Haikou, Hainan, China

**Keywords:** ferroptosis, tumor therapy, indole-3-aldehyde, AHR, c-JUN, DNA damage, *Lactobacillus reuteri*

## Abstract

Ferroptosis, a lipid peroxidation–driven form of regulated cell death, has emerged as a promising target for cancer therapy. However, the endogenous metabolic checkpoints restraining ferroptosis remain poorly defined. Here, we identify the tryptophan-derived indole metabolite, indole-3-aldehyde (I3A), as a potent suppressor of ferroptosis. Mechanistically, I3A activates the aryl hydrocarbon receptor to suppress c-Jun N-terminal kinase–c-JUN signaling under oxidative stress. This inhibition of c-JUN limits autophagic flux by downregulating LC3B expression, thereby stabilizing nuclear glutathione peroxidase 4. As a result, I3A not only prevents ferroptosis-associated lipid peroxidation but also mitigates oxidative DNA damage. In mouse models of melanoma and colorectal cancer, I3A administration significantly reduced the antitumor efficacy of the ferroptosis inducer RSL3, accompanied by reduced lipid peroxidation and preserved glutathione peroxidase 4 levels. Furthermore, gut colonization with Lactobacillus *reuteri* increased I3A concentration and conferred ferroptosis resistance *in vivo*. Together, these findings identify a host–microbe metabolic axis in which microbial I3A suppresses cancer cell ferroptosis through aryl hydrocarbon receptor–c-Jun N-terminal kinase signaling, which may have critical implications for redox-based cancer therapies.

Ferroptosis is a regulated, nonapoptotic, iron-dependent cell death characterized by excessive lipid peroxidation and a distinct redox-metabolic signature (1, 2). Ferrous iron catalyzes the formation of reactive oxygen species (ROS) *via* Fenton chemistry, amplifying membrane damage through uncontrolled phospholipid hydroperoxide generation. Central to ferroptosis resistance is glutathione peroxidase 4 (GPX4), an antioxidant enzyme that detoxifies lipid hydroperoxides and maintains cellular redox homeostasis (3, 4, 5). Among the established inducers of ferroptosis, erastin and RSL3 are the most widely studied small molecules. Erastin targets the cystine–glutamate antiporter system Xc^-^ (SLC7A11), leading to glutathione depletion and reduced activity of GPX4 (6). In contrast, RSL3 directly binds to and inhibits GPX4, thereby promoting the accumulation of lipid ROS and triggering ferroptotic cell death (7). Both compounds have demonstrated potent antitumor activity in preclinical studies by selectively killing cancer cells with high oxidative stress or impaired antioxidant defenses (8). Consequently, pharmacological induction of ferroptosis by erastin, RSL3, or related agents has emerged as a promising therapeutic strategy to overcome resistance to conventional chemotherapy and immunotherapy (9). Although the critical role of GPX4 is well established, the upstream metabolic and signaling networks governing its stability under ferroptotic stress remain incompletely understood.

Beyond membrane damage, ferroptosis has also been linked to genomic instability (10). Lipid peroxidation–associated ROS can diffuse into the nucleus and cause oxidative DNA damage, including strand breaks and base oxidation (11, 12). Emerging evidence implicates ferroptosis in the activation of DNA damage markers, such as γH2AX and 8-oxo-dG, along with disruption of DNA repair pathways (13, 14). These findings suggest that ferroptosis represents a dual threat to both membrane and nuclear integrity. However, the regulatory networks that shield both membranes and DNA from ferroptotic injury remain to be fully elucidated.

Tryptophan (Trp) metabolism has recently gained attention as a critical regulator of redox balance. Trp is primarily catabolized *via* three major enzymatic routes: the kynurenine pathway, initiated by indoleamine-2,3-dioxygenase 1–Trp-2,3-dioxygenase; the serotonin pathway; and the indole pathway, primarily catalyzed by interleukin-4-induced gene 1 (IL4I1). These pathways generate a diverse array of biologically active metabolites with known immunoregulatory and antioxidant properties (15, 16, 17, 18, 19, 20). Among them, indole-3-aldehyde (I3A) has been identified as a high-affinity ligand for the aryl hydrocarbon receptor (AHR), a redox-sensitive transcription factor and noncanonical E3 ubiquitin ligase that shapes immunity, xenobiotic defense, and oxidative stress responses (21, 22).

Previous studies, including ours, have highlighted the antitumor properties of I3A in diverse pathological settings by stimulating tumor immunogenicity or T-cell activity, suggesting its beneficial role in maintaining tissue homeostasis (13, 23). However, whether and how I3A regulates ferroptosis, particularly in the context of cancer therapy—has not been defined. The intestinal microbiota adds an additional layer of complexity: commensals such as *Lactobacillus reuteri* efficiently generate I3A, thereby extending the tumor-modulating effect of Trp metabolism from host to microbes (24). Whether microbiota-derived I3A regulates tumor sensitivity to ferroptosis-based therapy *in vivo* remains largely unexplored. These observations prompted us to investigate whether Trp-derived indole metabolites, particularly I3A, could influence ferroptosis sensitivity through AHR-dependent mechanisms.

In this study, we identified I3A as a potent endogenous ferroptosis inhibitor through a screen of Trp metabolites. Mechanistically, I3A engages AHR signaling to promote c-Jun N-terminal kinase (JNK) degradation, thereby repressing c-JUN-mediated transcription of LC3B and limiting autophagic degradation of GPX4. Stabilization of GPX4 preserves lipid peroxide detoxification and protects cells from ferroptotic DNA damage. Moreover, microbial production of I3A by *L*. *reuteri* strongly induces cancer cell resistance to ferroptosis-based cancer therapy in tumor-bearing mice. These findings define a host–microbe Trp–AHR–GPX4 axis that acts as a metabolic rheostat to restrain ferroptosis and may limit the efficacy of ferroptosis-based cancer therapies.

## Results

### IL4I1-derived I3A suppresses ferroptosis across diverse cell types

**Figure 1:**
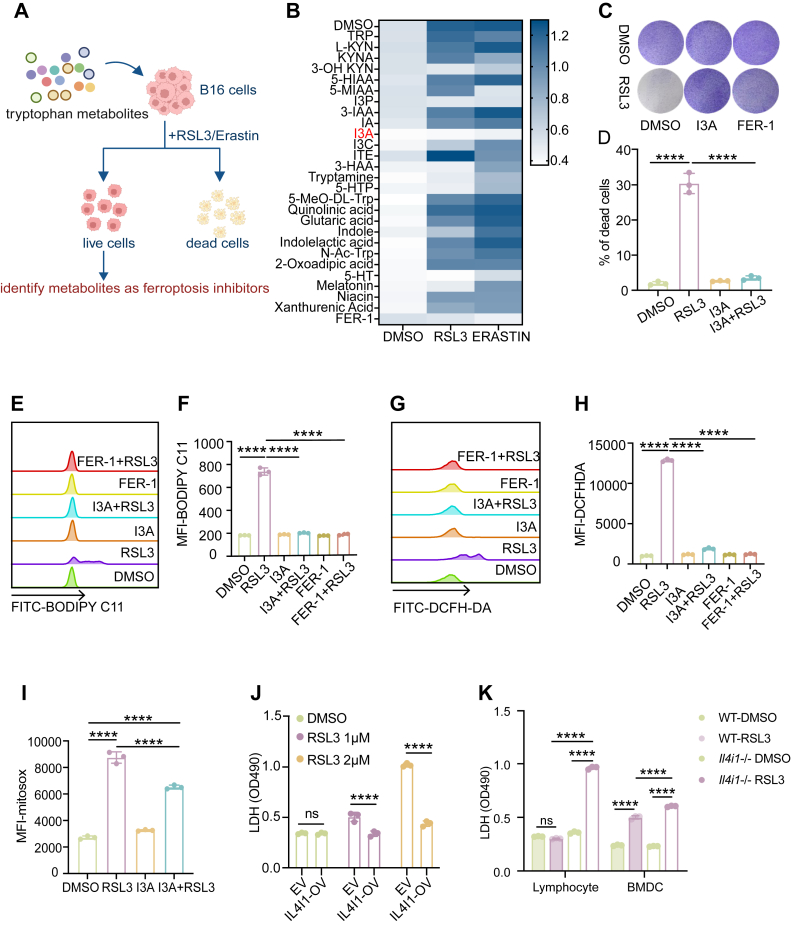
**IL4I1-derived I3A suppresses ferroptosis**. *A*, experimental workflow: a 26-compound Trp-metabolite library was screened in B16 cells to identify ferroptosis modulators. *B*, B16 cells were exposed to RSL3–erastin (1 μM) in conjunction with Trp metabolites (500 μM) for a duration of 24 h. Cell death was evaluated using an LDH assay, with the ferroptosis inhibitor Fer-1 (10 μM) employed as a positive control. *C*, *crystal violet* staining of viable B16 cells treated with I3A (500 μM) and RSL3 (1 μM) for 24 h. Fer-1 (10 μM) was employed as a positive control. *D*, B16 cells were treated with I3A (500 μM) and RSL3 (1 μM) for 8 h, and cell death was detected by FACS. *E*–*H*, B16 cells were treated with I3A (500 μM) and RSL3 (1 μM) for 3 h, cell peroxidation was detected by FACS, representative histograms are shown in (*E*, *G*), and the mean fluorescence ratio is shown in (*F*, *H*). *I*, B16 cells were treated with I3A (500 μM) and RSL3 (1 μM) for 3 h, and cell mitochondrial superoxide was detected by FACS, the mean fluorescence ratio is shown in *I*. *J*, B16 cells were transfected to overexpress EV or IL4I1, were treated with RSL3 (1 or 2 μM) for 24 h, and cell death was detected by LDH assay. *K*, BMDCs and lymphocytes from WT or *Il4i1*^−/−^ mice were treated with RSL3 (1 μM) for 24 h, and cell death was detected by LDH assay. Data are shown as mean ± SEM. Ns denotes not significant; ∗∗∗∗*p* < 0.0001. Statistical analysis was performed using one-way ANOVA with Bonferroni’s post-test for *D*–*K*. *B*–*K*, representative of at least three independent biological replicates. BMDC, bone marrow–derived dendritic cell; EV, empty vector; FACS, fluorescence-activated cell sorting; I3A, indole-3-pyruvic acid; IL4I1, interleukin-4-induced gene 1; LDH, lactate dehydrogenase; Trp, tryptophan.

To determine whether Trp-derived metabolites influence ferroptosis sensitivity, we screened a small library of 26 Trp metabolites in B16 cells (Fig. 1*A*). Through this screening process, we identified I3A, an indole-pathway metabolite, which significantly diminished the sensitivity of B16 cells to both RSL3 and erastin. Consistent with previous reports, we also identified ferroptosis-inhibitory effects for indole-3-pyruvic acid (I3P) and 5-hydroxytryptamine (17, 19), confirming the specificity and robustness of our screening approach (Fig. 1*B*). Subsequent validation experiments confirmed that I3A effectively inhibited RSL3-induced cell death across a range of tumor cell lines (Fig. S1, *A–D*). Cell viability assays further demonstrated that I3A substantially protected against RSL3-induced cytotoxicity (Fig. 1, *C* and *D*, Fig. S1*E*). To determine an *in vitro* concentration that achieves near-complete ferroptosis inhibition, we performed a dose–response analysis of I3A. Treatment with 500 μM I3A provided nearly complete protection against RSL3-induced ferroptosis and was therefore selected as the experimental concentration for subsequent *in vitro* assays (Fig. S1, *E–I*). Given that lipid peroxidation is a defining feature of ferroptosis, we next examined whether I3A modulates oxidative stress under ferroptotic conditions. Flow cytometry analysis revealed that I3A significantly attenuated RSL3-induced oxidative damage, as indicated by reduced lipid peroxidation, diminished intracellular ROS, and lower mitochondrial superoxide accumulation (Fig. S1, *F–I*).

Endogenous I3A production in mammalian cells is catalyzed by IL4I1, the key enzyme responsible for Trp conversion to I3A (19, 25). Thus, we hypothesized that alterations in IL4I1 expression might be associated with cellular sensitivity to ferroptosis by controlling endogenous I3A production. First, Pan-cancer transcriptomic analysis of The Cancer Genome Atlas revealed a positive correlation between IL4I1 and GPX4, a key ferroptosis suppressor (Fig. S1, *J–L*). To test a causal relationship between these two factors, we overexpressed IL4I1 in B16 cells. IL4I1 overexpression significantly suppressed RSL3-induced cell death, even in the absence of exogenous I3A (Fig. 1*J*, Fig. S1, *M* and *N*), supporting a cell-intrinsic role for IL4I1 in ferroptosis resistance. Notably, ferroptosis also affects nonmalignant cells (1). We observed that bone marrow–derived dendritic cells and splenic lymphocytes from *Il4i1* knockout (*Il4i1*^−/−^) mice exhibited increased sensitivity to ferroptosis (Fig. 1*K*). These results indicate that IL4I1-derived I3A functions as a general inhibitor of ferroptosis.

### AHR mediates I3A-induced ferroptosis inhibition

The structurally related metabolite I3P has been reported to inhibit ferroptosis *via* radical-trapping antioxidant activity independent of AHR signaling (20). To test whether I3A acts *via* a similar mechanism, we performed a 2,2-diphenyl-1-picrylhydrazyl (DPPH) radical scavenging assay. The data showed that I3A exhibited no intrinsic antioxidant activity (Fig. S2*A*), suggesting that it might inhibit ferroptosis *via* an indirect mechanism.

Previous studies have revealed that most indole metabolites derived from Trp act as endogenous ligands for AHR (26). Given that I3A is a high-affinity endogenous ligand of AHR (24), we hypothesized that I3A may mediate ferroptosis inhibition *via* AHR activation. RNA-Seq analysis of B16 cells treated with I3A revealed upregulation of canonical AHR target genes (Fig. S2*B*), indicating activation of the AHR pathway.

**Figure 2:**
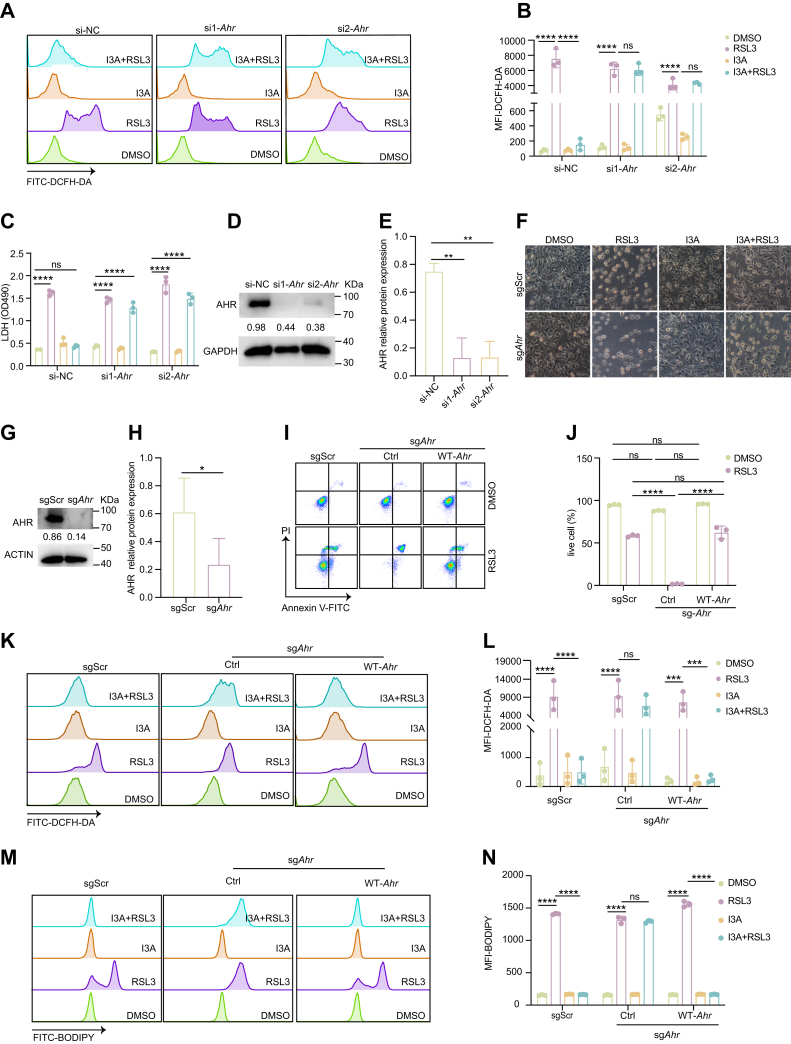
**AHR is required for the antiferroptotic activity of I3A**. *A* and *B*, B16 cells were transfected with negative control (NC) or si*Ahr* for 48 h, then treated with I3A (500 μM) or RSL3 (1 μM) for 3 h, and ROS accumulation was detected by FACS, representative histogram is shown in (*A*), and the mean fluorescence ratio is shown in (*B*). *C*, B16 cells were treated with I3A (500 μM) or RSL3 (1 μM) for 24 h, and cell death was detected by LDH assay. *D* and *E*, the expression of AHR following siRNA transfection was measured (*D*), and quantitative results are shown in I. *F*, B16 cells were treated with I3A (500 μM) or RSL3 (1 μM) for 24 h, and then cell morphology was examined. *G* and *H*, the knockout efficiency of AHR was detected (*G*), and quantitative results are shown in (*H*). *I* and *J*, B16 cells were treated with I3A (500 μM) or RSL3 (1 μM) for 12 h, cell viability and death were detected by FACS, representative histogram is shown in (*I*), and the mean fluorescence ratio is shown in (*J*). *K*–*N*, detected by FACS, representative histogram is shown in (*K*, *M*), and the mean fluorescence ratio is shown in (*L*, *N*). Data are shown as mean ± SEM. Statistical comparisons were performed using one-way or two-way ANOVA, followed by Dunnett’s multiple comparisons test for *B*–*N*. ns denotes not significant; ∗∗∗*p* < 0.001, ∗∗∗∗*p* < 0.0001. The results are representative of at least three independent biological replicates. AHR, aryl hydrocarbon receptor; FACS, fluorescence-activated cell sorting; I3A, indole-3-aldehyde; LDH, lactate dehydrogenase; ROS, reactive oxygen species.

Functionally, knockdown of AHR using siRNA markedly abolished the ferroptosis-inhibitory effect of I3A (Fig. 2, *A–E*). Consistently, CRISPR–Cas9-mediated Ahr knockout (sg*Ahr*) completely abrogated the ferroptosis-inhibitory ability of I3A (Fig. 2, *F–H*), and *Ahr*-deficient cells exhibited heightened sensitivity to RSL3 (Fig. 2, *I* and *J*). Notably, reconstitution with WT AHR into sg*Ahr* cells effectively restored I3A-mediated attenuation of oxidative stress and ferroptotic cell death (Fig. 2, *K–M*, Fig. S2, *C–E*), indicating that I3A-mediated ferroptosis inhibition is AHR dependent.

Furthermore, Cell Counting Kit-8 assays confirmed that I3P retained its ferroptosis-protective capacity in sg*Ahr* cells, whereas I3A did not (Fig. S2, *F–I*), highlighting a mechanistic divergence between these structurally related indole metabolites.

Collectively, our findings indicate that I3A suppresses ferroptosis through an AHR-dependent signaling mechanism rather than a direct radical-trapping antioxidant.

### The I3A–AHR axis restrains JNK–c-JUN signaling to inhibit ferroptosis

**Figure 3:**
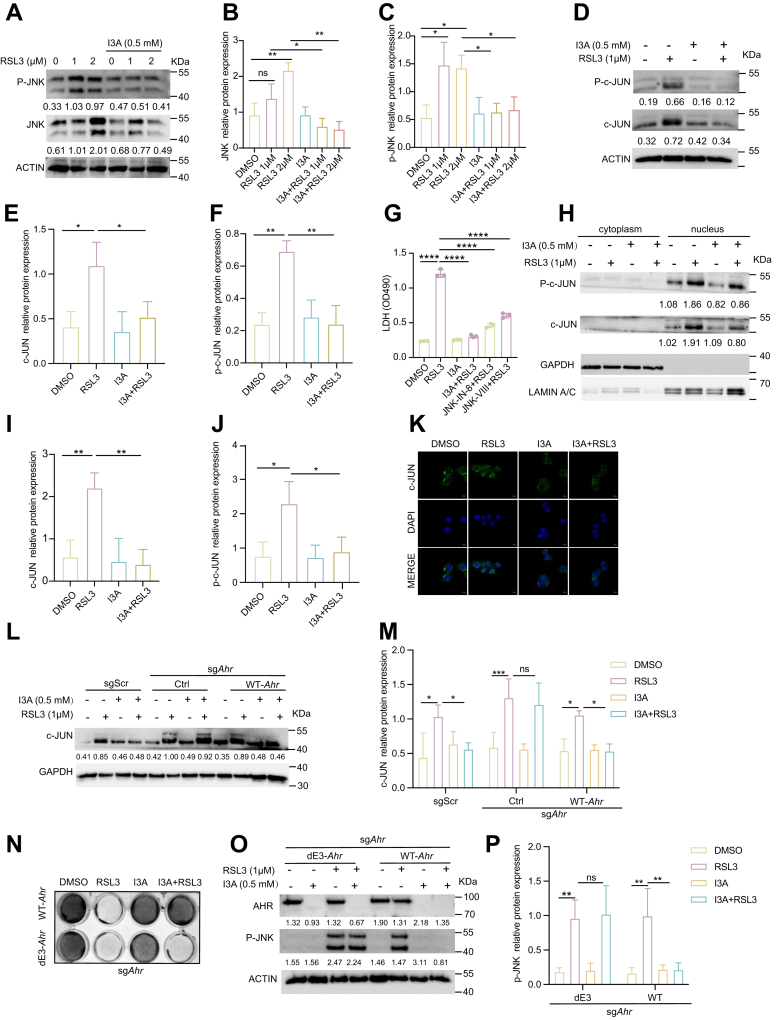
**The I3A–AHR axis restrains JNK–c-JUN signaling to inhibit ferroptosis**. *A*–*C*, B16 cells were treated with I3A (500 μM) or RSL3 (1 or 2 μM) for 3 h, the phospho-JNK and total JNK protein levels were detected (*A*), and quantitative results are shown in (*B* and *C*). *D*–*F*, B16 cells were treated with I3A (500 μM) or RSL3 (1 μM) for 3 h, the phospho-c-JUN and total c-JUN protein levels were detected (*D*), and quantitative results are shown in (*E* and *F*). *G*, B16 cells were treated with JNK-IN-8 or JNK-VIII (JNK–c-JUN inhibitors, 10 μM) or RSL3 (1 μM) for 24 h, and cell death was detected by LDH assay. *H*–*J*, B16 cells were treated with I3A (500 μM) or RSL3 (1 μM) for 3 h, nuclear and cytoplasmic protein levels of phospho-c-JUN and total c-JUN were detected (*H*), and quantitative results are shown in (*I* and *J*). *K*, B16 cells were treated with I3A (500 μM) or RSL3 (1 μM) for 2 h, the c-JUN protein levels and localization were detected by immunofluorescence assay, c-JUN (*green*) with DAPI nuclear counterstain (*blue*); the scale bar represents 10 μm. *L* and *M*, B16 cells were treated with I3A (0.5 mM) or RSL3 (1 μM) for 3 h, the c-JUN protein level was detected (*L*), and quantitative results are shown in (*M*). *N*, sg*Ahr* B16 cells re-expressing WT or De3-*Ahr* were treated with I3A (−0.5 mM) or RSL3 (1 mM) for 24 h, and cell viability was assessed using a crystal violet staining assay. *O* and *P*, B16 cells were treated with I3A (0.5 mM) or RSL3 (1 μM) for 3 h, phospho-JNK protein level was detected (*O*), and quantitative results are shown in (*P*). Data are shown as mean ± SEM. Ns denotes not significant; ∗∗∗∗*p* < 0.0001. Statistical analysis was performed using one-way or two-way ANOVA, followed by Dunnett’s multiple comparisons test for *B*–*P*. The results are representative of at least three independent biological replicates. AHR, aryl hydrocarbon receptor; DAPI, 4′,6-diamidino-2-phenylindole; I3A, indole-3-aldehyde; JNK, c-Jun N-terminal kinase; LDH, lactate dehydrogenase.

Given the critical role of AHR in I3A-mediated ferroptosis suppression, we next explored whether this protective effect involves modulation of key stress-response pathways. To test this, we systematically profiled a panel of stress-responsive signaling pathways, including AKT, PTEN, JNK–c-JUN, mTOR, p53, and HIF1a, all of which have been previously implicated in ferroptosis susceptibility (27, 28, 29, 30). Among these pathways examined, we found that I3A exhibited selective modulation of RSL3-induced signaling: it significantly attenuated JNK–c-JUN and HIF1α activation, while conversely restoring p53 expression suppressed by RSL3, with minimal effects on the remaining pathways (Fig. 3, *A–F*, Fig. S3, *A–K*).

Based on these observations, we next functionally validated the roles of JNK, HIF1a, and p53 signaling pathways that contributed to I3A–AHR-mediated ferroptosis inhibition. First, siRNA-mediated knockdown of p53 had no effect on I3A-mediated ferroptosis protection (Fig. S3, *L–N*). Similarly, HIF1a antagonist LW6 failed to rescue RSL3-mediated cell death (Fig. S3*O*). In striking contrast, JNK antagonists, JNK-VIII and JNK-IN-8, phenocopied the antiferroptotic effect of I3A (Fig. 3*G*), supporting a functional link between JNK and I3A in suppressing ferroptosis. We therefore investigated whether the I3A–AHR axis exerts its antiferroptotic effect through downregulating the RSL3-induced JNK–c-JUN activation. Transcriptomic profiling further revealed that RSL3-induced *c-Jun* expression was completely abrogated in the presence of I3A (Fig. S3*P*). Furthermore, at the protein level, both immunofluorescence and subcellular fractionation assays confirmed that I3A completely blocked RSL3-triggered c-JUN accumulation and nucleus translocation (Fig. 3, *H–K*). Importantly, this regulatory axis was strictly AHR dependent: I3A lost its c-JUN-suppressive effect in sg*Ahr* cells but fully regained it upon WT-AHR reconstitution (Fig. 3, *L–M*, Fig. S2*E*).

To elucidate the molecular mechanism by which I3A–AHR suppresses RSL3-induced JNK–c-JUN activation, we considered prior evidence that AHR can function as a noncanonical E3 ubiquitin ligase (31), and ubiquitin-mediated regulation of JNKc-JUN has been well documented (32, 33). We therefore hypothesized that AHR might exert its regulatory effect through this enzymatic activity. Consistent with this notion, reconstitution of sg*Ahr* cells with an E3 ligase–deficient AHR mutant failed to restore I3A-mediated suppression of JNK phosphorylation or ferroptotic resistance (Fig. 3, *N–P*), demonstrating that AHR’s E3 ligase function is critical for downstream inhibition of the JNK–c-JUN pathway. Furthermore, our prior study showed activation of an XRE-Luc reporter by I3A, indicating transcriptional activation of AHR (34). Consistently, the E3 ligase–deficient AHR mutant did not impair canonical transcriptional activity (Fig. S3*Q*). Collectively, these results identify JNK–c-JUN as a proferroptotic signaling module repressed by the I3A–AHR axis *via* E3 ligase–mediated JNK degradation.

Finally, to systematically exclude alternative mechanisms, we noted that *Hmox1*, a known dual regulator of ferroptosis (35, 36), was strongly induced by RSL3, which was largely reversed by I3A treatment (Fig. S3*P*). However, in this study, knockout of *Hmox1* expression *via* CRISPR–Cas9 technology failed to affect either RSL3-induced cell death or I3A-mediated protection (Fig. S3, *R* and *S*). These findings indicate that suppression of the JNK–c-JUN axis, rather than modulation of Hmox1 or other stress pathways, is the primary mechanism underlying I3A’s antiferroptotic activity.

### I3A preserves nuclear GPX4 by blocking c-JUN-mediated autophagic degradation

**Figure 4:**
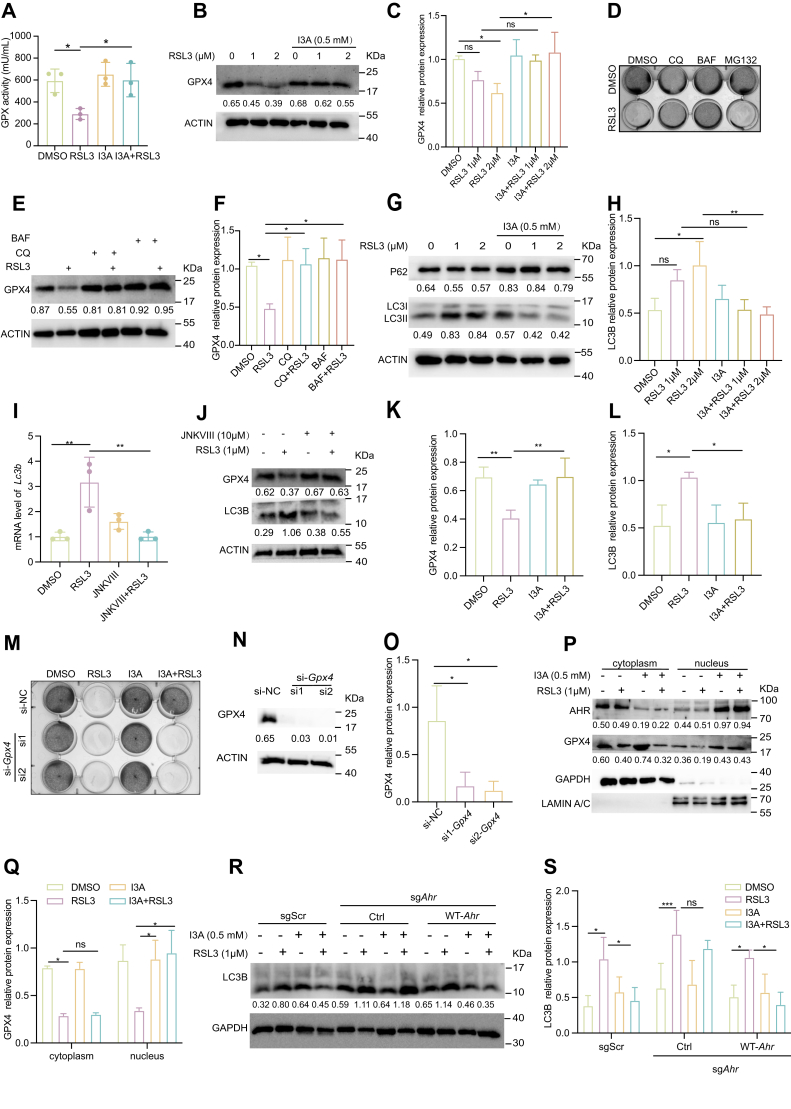
**I3A inhibits ferroptosis *via* the AHR–JNK–c-JUN–GPX4 signaling axis**. *A*, B16 cells were treated with I3A (0.5 mM) or RSL3 (1 μM) for 2 h, and then GPX activity was detected with a commercial kit. *B* and *C*, B16 cells were treated with I3A (0.5 mM) or RSL3 (1 μM) for 3 h, the expression of GPX4 was detected (*B*), and quantitative results are shown in (*C*). *D*, B16 cells were treated with different inhibitors or RSL3 (1 μM) for 24 h, and the cell viability was assessed using a crystal violet staining assay. Autophagy inhibitors include CQ (10 μM) and BAF (10 μM), and proteasome inhibitor includes MG132 (10 μM). *E* and *F*, B16 cells were treated with CQ, BAF, or RSL3 for 3 h, the levels of GPX4 were detected (*E*), and quantitative results are shown in (*F*). *G* and *H*, B16 cells were treated with I3A (0.5 mM) or RSL3 (1 mM) for 3 h, the levels of p62 and LC3 were detected by Western blot analysis (*G*), and quantitative results of LC3 are shown in (*H*). *I*, B16 cells were treated with JNK inhibitor JNK-VIII (10 μM) or RSL3 for 2 h, and mRNA expression of Lc3b was detected by quantitative PCR. *J*–*L*, B16 cells were treated with JNK-VIII (10 μM) or RSL3 (1 μM) for 3 h, the expression of GPX4 and LC3 was detected by Western blot analysis (*J*), and quantitative results are shown in *K* and *L*. *M*, B16 cells were transfected with siGpx4 or negative control (NC) for 48 h, then cells were treated with I3A (0.5 mM) or RSL3 (1 μM) for 24 h, and cell viability was assessed using a *crystal violet* staining assay. *N* and *O*, the expression of GPX4 following siRNA transfection in B16 cells was detected by Western blot analysis (*N*), and quantitative results are shown in (*O*). *P* and *Q*, B16 cells were treated with I3A (0.5 mM) or RSL3 (1 μM) for 3 h, and nuclear and cytoplasmic protein expressions of GPX4 were detected by Western blot analysis (*P*), and quantitative results are shown in (*Q*). *R* and *S*, B16 cells were treated with I3A (0.5 mM) or RSL3 (1 μM) for 3 h, and the expression of LC3 was detected by Western blot analysis (*R*), and quantitative results are shown in (*S*). Data were shown as mean ± SEM. Ns denotes not significant; ∗*p* < 0.05; ∗∗*p* < 0.01. Statistical analysis was performed using one-way or two-way ANOVA, followed by Dunnett’s multiple comparisons test. The results are representative of at least three independent biological replicates. AHR, aryl hydrocarbon receptor; BAF, bafilomycin; CQ, chloroquine; GPX4, glutathione peroxidase 4; I3A, indole-3-aldehyde; JNK, c-Jun N-terminal kinase.

Previous studies showed that RSL3 directly targets GPX4 to induce ferroptosis (7). As a terminal antioxidant defender against ferroptosis, GPX4 directly reduces phospholipid hydroperoxides to prevent ferroptosis (37). In this study, we observed that while RSL3 treatment did not alter *Gpx4* mRNA expression (Fig. S4), it markedly depleted GPX4 protein and abolished its enzymatic activity. Remarkably, both effects were potently reversed by I3A treatment (Fig. 4, *A–C*), suggesting that I3A stabilizes GPX4 at the protein level. We next investigated whether the I3A–AHR–JNK–c-JUN axis ultimately converges on GPX4 regulation.

To further elucidate the mechanistic basis of I3A-mediated GPX4 stabilization, we initially characterized RSL3-induced GPX4 degradation pathways. Previous studies have established that GPX4 homeostasis is coordinately regulated by both ubiquitin–proteasome and autophagy–lysosome pathways (7, 38); therefore, we compared the effects of proteasomal and lysosomal inhibitors on the protein stability of GPX4. Notably, only lysosomal inhibitors, chloroquine and bafilomycin A1, but not the proteasomal inhibitor MG132, prevented both RSL3-induced cell death and GPX4 depletion (Fig. 4, *D–F*), indicating that GPX4 is mainly degraded through autophagy rather than the proteasome pathway under ferroptotic stress. Further analysis revealed that RSL3 activated autophagic flux, as evidenced by increased LC3B lipidation and p62 degradation, both of which were reversed by I3A treatment (Fig. 4, *G* and *H*).

Building on our demonstration that I3A–AHR suppresses ferroptosis through JNK–c-JUN pathway inhibition, we next examined whether this signaling cascade mediates GPX4 autophagic degradation to drive ferroptotic cell death. Consistent with prior reports demonstrating that JNK-c-JUN-mediated transcriptional regulation of *Lc3b* (39), we found that pharmacological inhibition of JNK–c-JUN *via* JNK-VIII suppressed RSL3-induced LC3B mRNA levels (Fig. 4*I*). Concomitantly, JNK inhibition restored GPX4 protein expression and reduced LC3B accumulation (Fig. 4, *J–L*), establishing JNK–c-JUN as a master regulator of GPX4 autophagic degradation.

Finally, to validate the functional necessity of GPX4 in mediating I3A’s antiferroptotic activity, we generated GPX4 knockdown cells using GPX4-specific siRNA (si*Gpx4*). Loss of GPX4 completely abolished I3A’s protective effect against RSL3-induced ferroptosis (Fig. 4, *M–O*), further confirming GPX4 stabilization as the key downstream effector mediating antiferroptosis effect of I3A. Notably, I3A preferentially stabilized nuclear GPX4 (Fig. 4, *P–Q*). Importantly, I3A-inhibited RSL3-induced LC3B expression was strictly dependent on AHR expression (Fig. 4, *R* and *S*).

Together, these findings demonstrate that I3A-activated AHR suppresses JNK–c-JUN–LC3B axis, thereby preventing autophagic degradation of GPX4—particularly in the nucleus—to protect cells from ferroptosis.

### I3A protects cells from ferroptosis-induced DNA damage

Previous studies have shown that ferroptosis might also induce genomic instability through ROS-mediated DNA damage (40, 41). Given the role of GPX4 in limiting lipid peroxidation–driven genotoxicity (23) and our finding that I3A specifically stabilizes nuclear GPX4, we next investigated whether the antiferroptosis effect of I3A–AHR–JNK–c-JUN axis might extend to genomic integrity preservation through this nuclear GPX4 stabilization mechanism.

**Figure 5:**
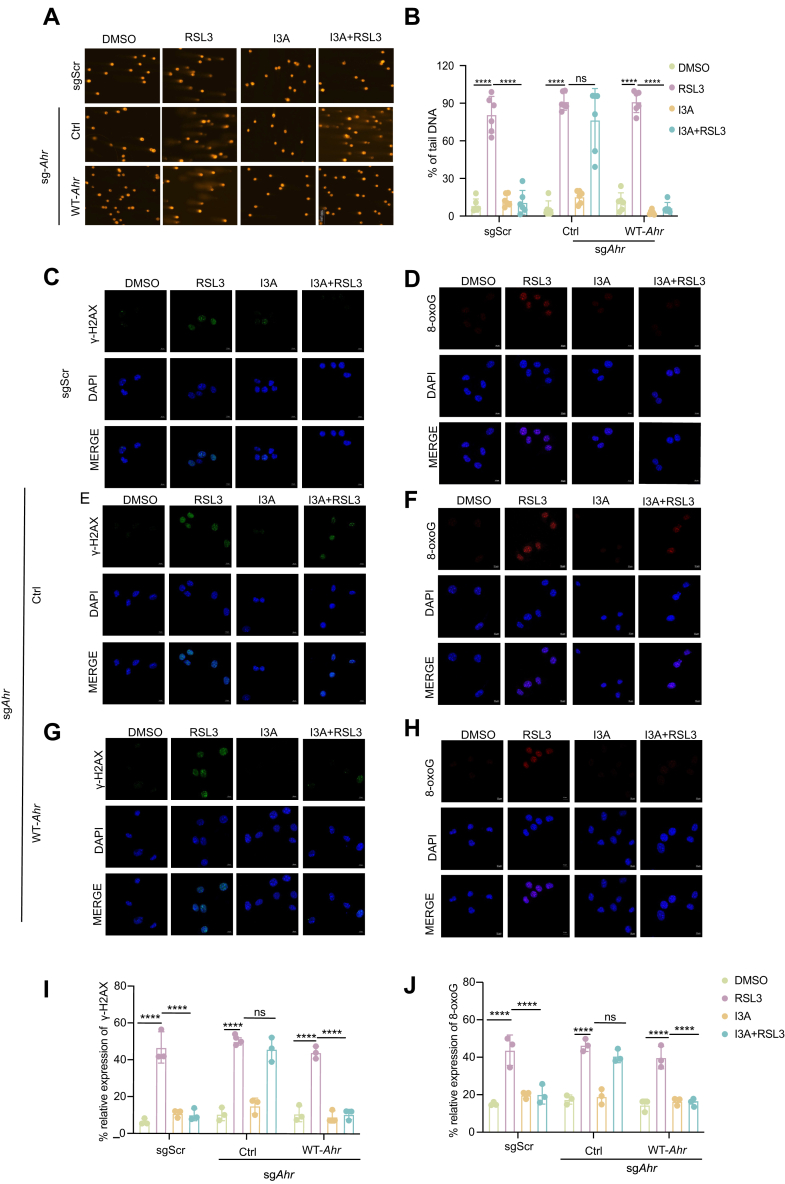
**I3A protects cells from ferroptosis-associated DNA damage**. *A* and *B*, B16 cells were treated with I3A (0.5 mM) or RSL3 (1 μM) for 3 h, DNA strand breaks were detected by alkaline comet assay (*A*), and quantitative results of the tail DNA are shown in (*B*). *C*–*J*, B16 cells were treated with I3A (0.5 mM) or RSL3 (1 μM) for 3 h, the expression of DNA damage markers, γH2AX and 8-oxoG, was detected by immunofluorescence assay (*C*–*H*), and the quantification of fluorescence intensity is shown in (*I*–*J*). Nuclei were counterstained with DAPI (*blue*). The scale bar represents 10 μm. Data are shown as mean ± SEM. Ns denotes not significant; ∗∗∗∗*p* < 0.0001. Statistical analysis was performed using two-way ANOVA, followed by Dunnett’s multiple comparisons test. The results are representative of at least three independent biological replicates. DAPI, 4′,6-diamidino-2-phenylindole; I3A, indole-3-aldehyde.

Using the alkaline comet assay, a sensitive method to measure DNA damage in individual cells (42), we observed extensive DNA fragmentation following RSL3 treatment, as evidenced by significantly elongated comet tails. This genotoxic effect was substantially attenuated by I3A cotreatment (Fig. 5, *A* and *B*). To further characterize the nature of this DNA damage, we stained the cells with two additional markers: γH2AX, a well-established indicator of DNA double-strand breaks (43), and 8-oxoG, a specific marker for oxidative DNA lesions resulting from lipid peroxidation byproducts (44). Immunofluorescence analysis demonstrated that both DNA damage markers were markedly elevated in RSL3-treated cells, and this induction was effectively suppressed by I3A in an AHR-dependent manner (Fig. 5, *C–J*).

Collectively, these findings define an I3A–AHR–JNK–c-JUN–GPX4 signaling axis that safeguards genomic integrity by stabilizing GPX4 and preventing ferroptosis-associated DNA damage.

### I3A compromises ferroptosis-based tumor therapy *in vivo*

**Figure 6:**
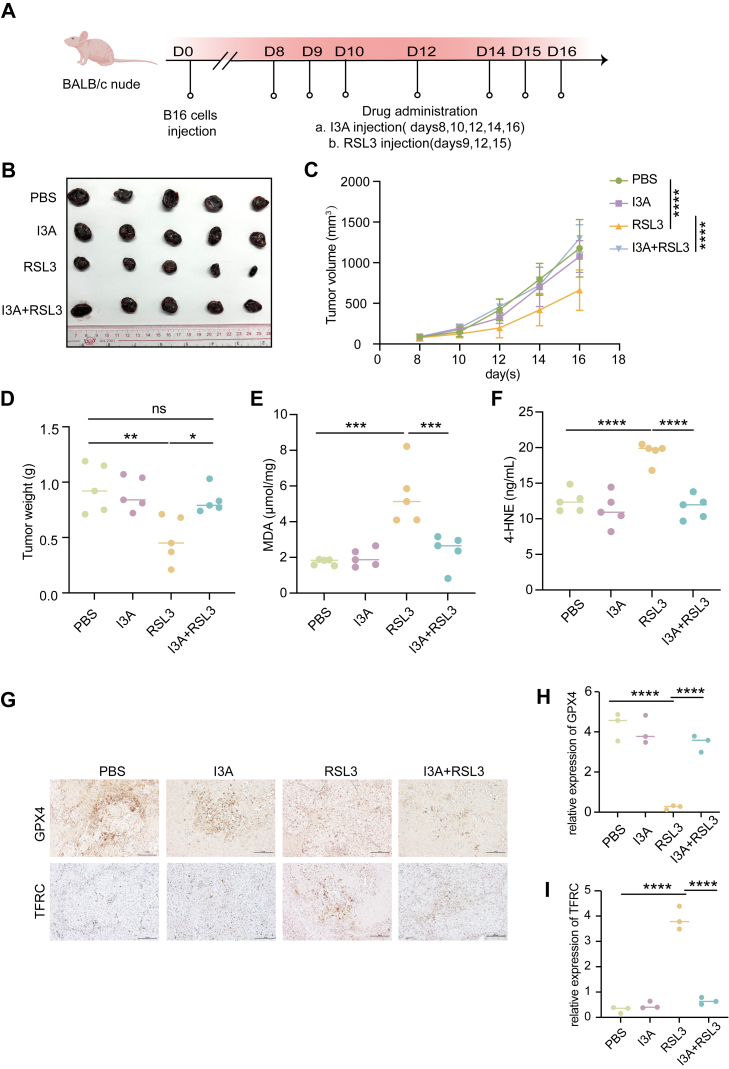
**I3A antagonizes RSL3-induced ferroptosis and tumor suppression *in vivo***. *A*, schematic illustration of the *in vivo* experimental design on the mouse tumor model; B16 cells (0.5 × 10^6^ per mouse) were subcutaneously inoculated on BALB/c-nude mice (day 0), following by intraperitoneal (i.p.) injection of RSL3 (50 mg kg^−1^) or vehicle solvent in combination with I3A (50 mg kg^−1^) or vehicle solvent on the indicated days, n = 5 in each group. *B*–*D*, mice were i.p. injected with I3A/vehicle in combination with RSL/vehicle, tumor growth of the mice was monitored and recorded every other day, then the tumor images (*B*), tumor growth curves (*C*), and final tumor weights (*D*) were shown. The endpoint is when the tumor volume reached 2000 mm^3^. *E* and *F*, at the endpoint, tumors were isolated, and quantification of lipid peroxidation markers, MDA and 4-HNE, was detected with commercially available kits. *G*–*I*, GPX4 and TFRC from tumors were detected by immunohistochemistry assay (*G*), the scale bar represents 100 μm, and the quantification of GPX4 and TFRC immunoreactivity (H-score) is shown in (*H* and *I*). Data are shown as mean ± SEM; each dot corresponds to one biological replicate. Ns denotes not significant; ∗*p* < 0.05; ∗∗*p* < 0.01; ∗∗∗*p* < 0.001; ∗∗∗∗*p* < 0.0001. Statistical analysis was performed using one-way ANOVA, followed by Tukey’s multiple comparisons test. GPX4, glutathione peroxidase 4; 4-HNE, 4-hydroxynonenal; I3A, indole-3-aldehyde; MDA, malondialdehyde; TFRC, transferrin receptor.

To assess the physiological relevance of I3A-mediated ferroptosis suppression, we next evaluated its effect *in vivo* using murine tumor models. Mice bearing subcutaneous B16 melanoma tumors were treated with the ferroptosis inducer RSL3, either alone or in combination with I3A (Fig. 6*A*).

In B16 melanoma tumors, treatment with the ferroptosis inducer RSL3 monotherapy markedly inhibited tumor growth, consistent with its established ferroptosis-inducing activity. However, coadministration of I3A treatment partially abrogated this therapeutic effect (Fig. 6, *B–D*). Consistent with these findings, in MC38 colorectal tumors, RSL3 treatment markedly inhibited tumor progression, whereas concomitant administration of I3A mitigated this antitumor response (Fig. S5, *A–D*). At the molecular level, previous studies have identified that malondialdehyde (MDA) and 4-hydroxynonenal (4-HNE) as major end products of lipid peroxidation and reliable biochemical indicators of ferroptotic oxidative damage (45). In addition, the transferrin receptor (TFRC), a key regulator of cellular iron uptake, has been recognized as a specific ferroptosis marker (46). Consistent with these findings, tumors from RSL3-treated mice exhibited pronounced increases in MDA and 4-HNE levels (Fig. 6, *E* and *F*, Fig. S5, *E* and *F*), along with loss of GPX4 and upregulation of TFRC (Fig. 6, *G–I*, Fig. S5, *G–I*). Importantly, coadministration of I3A reversed these ferroptotic alterations, indicating that I3A effectively antagonizes ferroptosis within the tumor microenvironment.

Together with the phenotypic observations described above, these results demonstrate that I3A suppresses ferroptosis-based therapy across distinct tumor types, underscoring a conserved metabolic checkpoint that can undermine the clinical efficacy of ferroptosis inducers.

### *L*. *reuteri* blunts ferroptosis-based therapy by supplying microbial I3A

Given that probiotic bacterium, such as *L*. *reuteri*, produces I3A through Trp metabolism (24, 47), we investigate whether *L*. *reuteri* could similarly modulate ferroptosis sensitivity *in vivo*.

**Figure 7:**
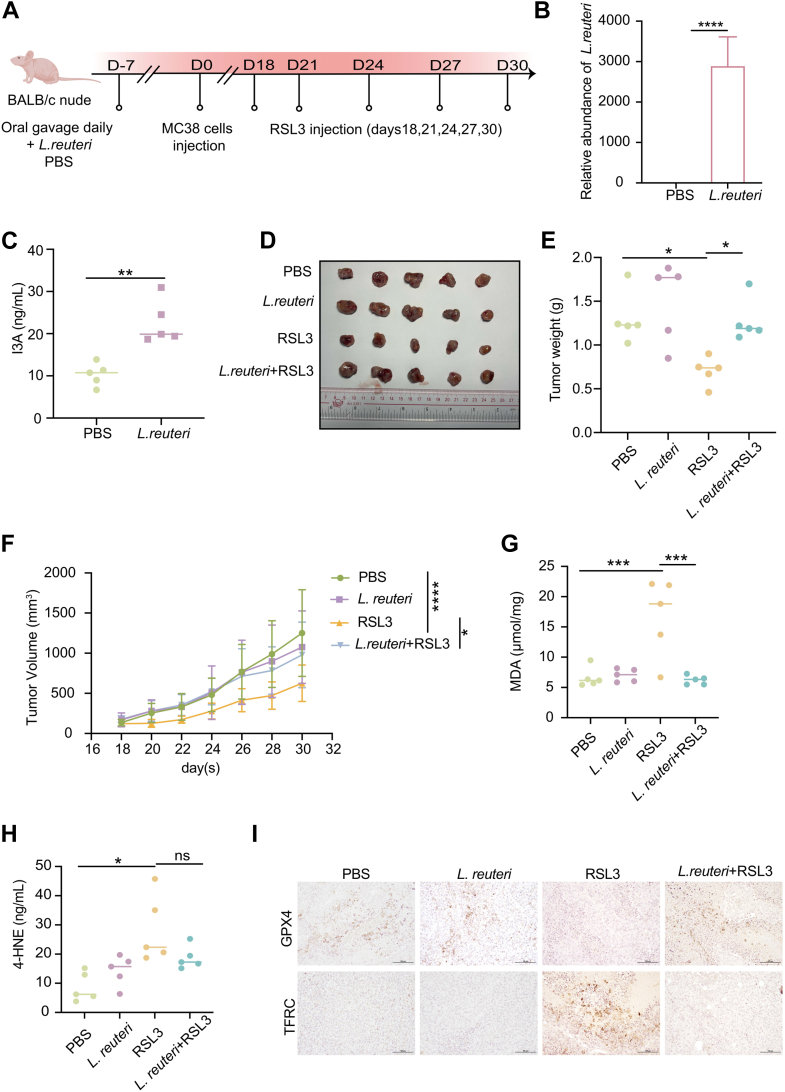
***Lactobacillus reuteri*–derived I3A attenuates RSL3-induced ferroptosis in MC38 tumors**. *A*, schematic illustration of the *in vivo* experimental design on the mouse colorectal tumor model; BALB/c nude mice were orally gavaged daily with PBS or *L*. *reuteri* (1 × 10^10^ colony-forming unit [CFU]) beginning 7 days before MC38 tumor implantation. MC38 cells (1 × 10^6^ per mouse) were then injected subcutaneously (day 0), followed by RSL3 (50 mg kg^−1^) administration as indicated. n = 5 in each group. *B*, at the endpoint, fecal samples were collected, and total bacterial DNA was extracted for quantitative PCR analysis. The relative abundance of *L*. *reuteri* was determined using species-specific primers and normalized to the PBS-treated group. *C*, serum of mice was collected, and the serum I3A relative concentrations were quantified by targeted LC–MS/MS. *D*–*F*, tumor growth of mice was monitored and recorded every other day, then the tumor images (*D*), tumor weight (*E*), and tumor growth curves (*F*) were shown, the endpoint is when the tumor volume reached 2000 mm^3^. *G* and *H*, at the endpoint, tumors were isolated, and quantification of lipid peroxidation markers, MDA and 4-HNE, isolated from tumors was detected with commercially available kits. *I*, GPX4 and TFRC from tumors were detected by immunohistochemistry assay; the scale bar represents 100 μm. Data are shown as mean or mean ± SEM; each dot corresponds to one biological replicate. ns denotes not significant; ∗*p* < 0.05; ∗∗*p* < 0.01; ∗∗∗*p* < 0.001; ∗∗∗∗*p* < 0.0001. Statistical analysis was performed using one-way ANOVA, followed by Tukey’s multiple comparisons test. GPX4, glutathione peroxidase 4; I3A, indole-3-aldehyde; TFRC, transferrin receptor.

In a probiotic colonization model, BALB/c nude mice bearing MC38 tumors received daily oral gavage of *L*. *reuteri* throughout the experimental period, including during tumor implantation and RSL3 treatment (Fig. 7*A*). This continuous administration significantly increased serum I3A concentrations, indicating systemic upregulation of the bacterial metabolite (Fig. 7, *B* and *C*). In this MC38 tumor model, treatment with RSL3 robustly suppressed tumor growth, whereas concomitant *L*. *reuteri* significantly blunted this antitumor efficacy (Fig. 7, *D–F*). Furthermore, tumors from *L*. *reuteri*-treated mice exhibited decreased accumulation of lipid peroxidation end products, including MDA and 4-HNE (Fig. 7, *G* and *H*). Consistently, *L*. *reuteri* restored GPX4 expression and reduced the ferroptosis-associated upregulation of TFRC expression in tumor tissues (Fig. 7*I*), thereby recapitulating the biochemical fingerprint of I3A treatment.

Taken together, these findings demonstrate that *L*. *reuteri*-generated I3A can attenuate RSL3-induced ferroptosis and weaken ferroptosis-based cancer therapy *in vivo*.

## Discussion

Emerging evidence highlights the significant role of gut microbiota–derived metabolites, particularly Trp derivatives, in regulating tumor initiation and progression (48, 49). Notably, indole-containing Trp metabolites have been implicated in modulating ferroptosis—a process increasingly recognized for its relevance in tumorigenesis (15). However, a critical unanswered question remains whether pharmacological blockade of these microbial metabolite–mediated ferroptosis protective mechanisms could enhance tumor sensitivity to ferroptosis-inducing therapies.

In this study, we identify I3A, an endogenous indole metabolite, as a potent suppressor of ferroptosis. Previous studies have demonstrated that intratumoral *L*. *reuteri*–derived I3A enhances immune checkpoint blockade efficacy through an AHR-dependent mechanism that expands interferon-γ-producing CD8^+^ T cells (24). Complementing these findings, our prior work revealed that I3A promotes tumor immunogenicity *via* downregulation of AHR and c-Myc signaling pathways (34). In contrast to these known immunostimulatory effects, in this study, we found that I3A strongly protects tumors from ferroptosis in immunodeficient models and *L*. *reuteri*–colonized systems, indicating that the functional outcomes of I3A are highly context dependent. We propose that the balance between these opposing activities–immunostimulation *versus* cytoprotection–likely depends on host immune status, microbiota composition, specific tumor microenvironment interactions, and relative activation of AHR pathways.

Under conditions of RSL3-induced oxidative stress, I3A engages the E3 ligase activity of AHR to selectively suppress JNK–c-JUN signaling pathway. Extensive literature indicates that although AHR serves as a common receptor for various Trp metabolites, its downstream effects display remarkable ligand specificity. For example, trans-3-indoleacrylic acid acts as an endogenous ligand of AHR that transcriptionally upregulates downstream genes to inhibit ferroptosis (15). By contrast, l-kynurenine and I3P have been reported to regulate ferroptosis through AHR-independent mechanisms (18, 19, 50). These observations suggest that the ability to modulate ferroptosis *via* AHR activation may be restricted to particular structural classes of Trp metabolites.

Our study elucidated a novel I3A–AHR–JNK–c-JUN signaling axis that stabilizes nuclear GPX4 protein by inhibiting its autophagic degradation, thereby preserving lipid peroxide detoxification and protecting against ferroptosis-associated DNA damage. While the antioxidant function of GPX4 is well established in cytoplasmic compartments (51, 52), our data reveal a preferential stabilization of the nuclear pool, raising a potential compartmentalized GPX4 activity in genomic protection against oxidative stress. This nuclear accumulation of GPX4 raises important questions regarding its specific roles in maintaining nuclear antioxidant functions, particularly in protecting genomic DNA from ferroptosis-induced oxidative damage. Further investigation will be required to determine whether nuclear GPX4 actively participates in redox homeostasis or serves alternative functions in this subcellular localization.

There are several limitations of this study. First, we did not define how I3A’s dual activities—enhancing antitumor immunity, yet suppressing ferroptosis—are quantitatively balanced within an intact immune microenvironment, which will require further studies using immune-competent models and across microbiota contexts in the future. Second, although we have implicated *L*. *reuteri*–derived I3A in ferroptosis resistance, we could not exclude the possibility that other bacterial metabolites may also contribute to this phenotype. Future study using untargeted metabolomics coupled with functional validation will be needed to identify potential coregulators. Finally, our *in vitro* assays employed relatively high I3A concentrations compared with basal levels of mouse serum I3A *in vivo*, and we did not measure the actual intracellular and intratumoral levels of I3A under basal or microbiota-supplemented conditions. Therefore, future pharmacokinetic and distribution studies will be necessary to formally refine the physiological and therapeutic relevance of these findings.

In summary, we have identified a Trp–AHR–GPX4 regulatory axis that coordinately protects cellular and genomic integrity during ferroptotic stress. Targeting this metabolic circuit may improve the antitumor efficacy of ferroptosis-based therapies and provide promising combinatorial strategies involving diet, microbiome, and redox metabolism. Overall, our work broadens the mechanistic framework of ferroptosis regulation by revealing a Trp metabolite–regulated nuclear GPX4 stabilization and highlights new targets for redox-oriented precision therapy.

## Experimental procedures

### Mice, cells, and reagents

Six- to 8-week-old female BALB/c nude mice were purchased from the Vital River Laboratory. *Il4i1*^−/−^ mice were purchased from GemPharmatech, Inc.

B16, human embryonic kidney 293T cells were purchased from the American Type Culture Collection, MC38 cells were kindly gifted by Dr Xuanming Yang (Shanghai Jiaotong University), LS174T, ID8, HCT116 cells were gifted by Dr Jing Tan (Sun Yat-Sen University Cancer Center), and A375 cell line was gifted by Dr Shuai Chen (Sun Yat-Sen University Cancer Center). All cell lines were routinely tested as being mycoplasma free. The cells were maintained either with Dulbecco’s modified Eagle's medium (Invitrogen) supplemented with 10% fetal bovine serum and 1% penicillin–streptomycin or RPMI1640 (Invitrogen) supplemented with 1% penicillin–streptomycin and 10% fetal bovine serum in a humidified atmosphere at 37 °C and 5% CO_2_.

The antibodies used for Western blot analysis, immunofluorescence, and immunohistochemistry staining include AHR (Santa Cruz; catalog no.: sc-133088), GPX4 (Abcam; catalog no.: ab125066), JNK and Phospho-JNK (CST; catalog no.: 9252/4668), c-JUN and Phospho-JUN (CST; catalog no.: 9165/3270), LC3B (Sigma; catalog no.: L8918), SQSTM1/p62 (CST; catalog no.: 39749s), AKT and Phospho-AKT(Ser473) (CST; catalog no.: 2920/4060), mTOR (CST; catalog no.: 2983), p53 (CST; catalog no.: 2524s), PTEN (CST; catalog no. 9188), HIF1A (CST; catalog no.: 36169), TFRC (Abcam; catalog no.: ab218544), ACTIN (Santa Cruz; catalog no.: sc8432), GAPDH (Santa Cruz; catalog no.: sc32233), phospho-Histone H2A.X (Ser139) (CST; catalog no.: 9718), and 8-oxoG DNA Lesion Antibody (Santa Cruz; catalog no.: sc130814). All antibodies were validated by the suppliers and used at dilutions recommended in the respective datasheets.

### Bacteria culture

*L*. *reuteri* was purchased from American Type Culture Collection (catalog no.: 23272) and cultured in MRS broth (BD Difco; catalog no.: DF0881-17-5) at 37 °C. The medium was centrifuged at 4000*g* for 10 min and then washed and resuspended in PBS for gavage.

### Tumor xenograft model

For the *in vivo* study, B16 tumor cells (0.5 × 10^6^ cells per mouse) or MC38 tumor cells (1 × 10^6^ cells per mouse) were subcutaneously injected into the right flank of nude mice. The mice were administered I3A (50 mg/kg), RSL3 (50 mg/kg) or PBS by intraperitoneal injection on the indicated time points. The tumor volume was calculated using the formula 0.5 × tumor length × (tumor width) (2), where the longer dimension was considered as the tumor length.

### Western blot

Cells were lysed using a lysis buffer supplemented with the PhosSTOP phosphatase inhibitor cocktail (Roche Diagnostics) and 1 mM DTT. Whole cell proteins were extracted by centrifugation at 12,000*g* for 8 min, with all procedures conducted on ice to maintain protein integrity. The extracted proteins were resolved *via* SDS-PAGE and subsequently transferred onto a polyvinylidene difluoride membrane. The membrane was blocked with 5% nonfat milk for 1 h at room temperature to prevent nonspecific binding. The membrane was incubated with the primary antibody overnight at 4 °C, followed by three washes with PBS with Tween-20, then incubated with the secondary antibody for 1 h at room temperature. Finally, the membrane was washed and subjected to chemiluminescence analysis using an ECL detection kit (Thermo Scientific; catalog no.: 32106).

### Immunofluorescence

Immunofluorescence imaging was conducted using a ZEISS LSM880 confocal microscope. Cells were seeded in a confocal cell culture dish (SPL Life Sciences; catalog no.: 200350) and maintained overnight at 37 °C. Subsequently, the cells were washed with PBS and fixed with 4% paraformaldehyde for 10 min. Following fixation, the cells were permeabilized with 0.2% Triton X-100 in PBS for 10 min and subjected to three PBS washes. F-actin was then stained with rhodamine–phalloidin reagent (Cytoskeleton, PHDR1, 100 nmol/l in PBS) for 1 h. Nuclei were stained with 4′,6-diamidino-2-phenylindole. Samples were mounted with antifade medium and imaged using a fluorescence or a confocal microscope. Image analysis was done with the ImageJ (National Institutes of Health,Bethsda) software.

### Immunohistochemistry

Immunohistochemistry staining was conducted to detect target protein expression and localization in tissue sections. After deparaffinization and rehydration, antigen retrieval was performed using Tris–EDTA buffer. Endogenous peroxidase was blocked with hydrogen peroxide, followed by serum blocking. Sections were incubated overnight at 4 °C with primary antibodies (GPX4 and TFRC [1:200 dilution]) and then with horseradish peroxidase–conjugated secondary antibodies at room temperature. 3,3´-Diaminodbenzidine substrate developed the signal, and hematoxylin counterstained the nuclei. Sections were then dehydrated, mounted, and imaged with a light microscope.

### Comet assay

Cells were mixed with low–melting-point agarose and layered onto a microscope slide precoated with normal–melting-point agarose. After solidification, slides were immersed in lysis buffer (2.5 M NaCl, 100 mM EDTA, 10 mM Tris, 1% Triton X-100, and 10% dimethyl sulfoxide, pH 10) at 4 °C for at least 1 h. DNA was unwound in alkaline electrophoresis buffer (300 mM NaOH, 1 mM EDTA, pH >13) for 20 min, followed by electrophoresis at 25 V (∼300 mA) for 20 to 30 min at 4 °C. Slides were then neutralized, fixed in ethanol, dried, and stained with a fluorescent DNA dye (*e*.*g*., SYBR Green or ethidium bromide). Comets were visualized using a fluorescence microscope, and DNA damage was quantified using OpenComet (OpenComet Project).

### CRISPR–Cas9 knockout and siRNA knockdown

*Ahr* and *Hmox1* knockout cells were generated utilizing the CRISPR–Cas9 technology as previously described (53). The single-guide RNA (sgRNA) sequences were designed using the Optimized CRISPR Design tool (available at http://chopchop.cbu.uib.no/). The guide sequences are listed in Table S1. The sgRNAs were cloned into the LentiCRISPR v2 vector, which also harbors the *Streptococcus pyogenes* Cas9 nuclease gene. The sgRNA lentiviral vectors were cotransfected with the pspax2 and pMD2.G packaging plasmids into 293T cells. Supernatants were collected 48 h post-transfection and subsequently used to infect tumor cells. Following infection, cells were selected with puromycin for 48 h. The knockout effect was verified through Western blot analysis of whole-cell protein extracts.

For the purpose of siRNA knockdown, B16 cells in the exponential growth phase were seeded into 6-well plates and subjected to transient transfection using siRNA and the RNAiMAX Transfection Reagent (Invitrogen; catalog no.: 13778150), following the manufacturer’s protocol. The sequences of the siRNAs used are listed in Table S1.

### RNA isolation and quantitative real-time PCR

Total RNA was extracted using Trizol reagent (Invitrogen; catalog no.: 15596018) in accordance with the manufacturer’s protocol. Subsequent reverse transcription of RNA was conducted employing a specific primer set and a reverse transcriptase reagent kit, which included a genomic DNA eraser. Quantitative real-time PCR (qPCR) was performed using the 2× PolarSignal SYBR Green Mix Taq (MIKX; catalog no.: MKG900-01) and analyzed with a Bio-Rad CFX96 thermal cycler. The sequences of the primers are listed in Table S2.

### Separation of cytoplasmic and nuclear fractions

Cytoplasmic and nuclear proteins were extracted following the manufacturer’s instructions (Beyotime; catalog no.: P0027). Briefly, cells were harvested and washed twice with ice-cold PBS, then resuspended in cytoplasmic extraction buffer containing protease and phosphatase inhibitors. After incubation on ice for 10 min, samples were vortexed briefly and centrifuged at 12,000*g* for 5 min at 4 °C to collect the cytoplasmic fraction (supernatant). The remaining pellet was lysed in nuclear extraction buffer on ice for 30 min with intermittent vortexing, followed by centrifugation at 12,000*g* for 10 min at 4 °C to obtain the nuclear fraction.

### GPX activity assay

GPX activity was measured according to the manufacturer’s instructions (Abcam; catalog no.: ab102530). Briefly, cells or tissue lysates were prepared on ice and centrifuged to remove debris. The supernatants were incubated with the reaction mixture containing glutathione, glutathione reductase, and NADPH. GPX activity was determined by monitoring the decrease in NADPH absorbance at 340 nm using a microplate reader (BioTek Synergy HT). The results were normalized to protein concentration and expressed as units per milligram of protein. All measurements were performed in at least three independent biological replicates.

### DPPH assay

The antioxidant capacity of the indicated compounds was determined by the DPPH radical scavenging assay. Briefly, metabolites were incubated with 0.2 mM DPPH solution (MedChemExpress; catalog no.: HY-112053) in ethanol for 30 min at room temperature in the dark, and absorbance at 517 nm was recorded using a microplate reader. The reduction in absorbance reflected the DPPH radical scavenging activity.

### Cell death, viability, and ROS assays

Cell death, viability, and ROS levels were assessed using multiple complementary approaches.

For the lactate dehydrogenase release assay, tumor cells were treated with the indicated compounds or drugs for the specified durations. The culture supernatants were collected, centrifuged to remove debris, and analyzed using a Cytotoxicity Assay Kit (Promega; catalog no.: G1780) following the manufacturer’s protocol. The absorbance at 490 nm was measured, and absorbance values were directly used to represent lactate dehydrogenase release levels.

For crystal violet staining, after removal of the culture supernatant, adherent cells were fixed with 4% paraformaldehyde for 5 min and stained with 0.5% crystal violet for 5 min at room temperature. Excess dye was removed by extensive rinsing with distilled water, after which the plates were air dried and imaged to visualize viable adherent cells.

For flow cytometric detection of cell death and viability, mixed cell populations were collected and stained with either Zombie NIR Fixable Viability Dye (BioLegend; catalog no.: 423105) or Annexin V/propidium iodide staining solution (Biosciences; catalog no.: B556547) according to the manufacturer’s instructions.

For ROS measurement, cells were washed twice with PBS and incubated with either DCFH-DA (Beyotime; catalog no.: S0033S) or BODIPY-C11 (Thermo Fisher; catalog no.: D3861) for 30 min at 37 °C in the dark. After staining, the dye was removed by washing, and cells were resuspended in 300 μl PBS for flow cytometric analysis.

The stained cells were analyzed by flow cytometry using a BD LSRFortessa X-20 cytometer. Data were analyzed using FlowJo (BD Biosciences), version 10.0. All experiments were performed in at least three independent biological replicates.

### Luciferase reporter assay

To evaluate the transcriptional activity of AHR, an XRE-Luc reporter assay was performed. Cells were seeded in 24-well plates and transfected with 500 ng of XRE-Luc reporter plasmid and 25 ng of Renilla TK control plasmid using Lipofectamine 2000 (Invitrogen) in Opti-MEM (Gibco) according to the manufacturer’s instructions. For rescue experiments, *Ahr*-knockout (sg*Ahr*) B16 cells were cotransfected with plasmids encoding WT AHR, the E3-ligase–deficient mutant AHR (Δ523–583) (6, 54) or an empty vector control. After 6 to 8 h, the medium was replaced with fresh complete medium. Twenty-four hours post-transfection, cells were treated with I3A or vehicle for an additional 24 h. Luciferase activity was measured using the Dual-Luciferase Reporter Assay System (Promega; catalog no.: E1910) following the manufacturer’s protocol. The full coding sequence of the E3-ligase–deficient AHR construct is provided in Table S3.

### Metabolite concentration analysis

To quantify serum concentrations of I3A in mice, 100 μl of serum from mice was extracted with 900 μl methanol, the mixture was vortexed at room temperature for 10 min, and centrifuged at 15,000*g* for 10 min at 4 °C. The supernatant (900 μl) was transferred to a new tube and centrifuged again under the same conditions and dried to dryness. The dried residues were reconstituted in 100 μl methanol and subjected to UPLC–electrospray ionization–MS/MS analysis as described previously (34).

### Mouse fecal DNA extraction and qPCR detection

Mouse feces were collected and stored at −80 °C for future use in DNA extraction and analysis. Genomic DNA was extracted using the QIAamp PowerFecal Pro DNA Kit (QIAGEN; catalog no.: 51804) according to the manufacturer’s protocol. Approximately 250 mg of fecal material was homogenized in CD1 buffer using a bead mill, followed by sequential centrifugation, washing, and elution steps as described by the kit manual. DNA was eluted in 50 μl of C6 buffer, quantified by NanoDrop spectrophotometry, and used for qPCR detection of *L*. *reuteri* abundance. The specific primer sequences used for qPCR are provided in Table S2.

### Study approval

All mice were maintained under specific pathogen-free conditions and in accordance with the animal experimental guidelines of Sun Yat-sen University (Guangzhou, China). All animal procedures were approved by the Institutional Animal Care and Use Committee of Sun Yat-sen University (approval number: 2022000536). The key raw data of this study have been deposited to the public platform (www.researchdata.org.cn) with the approval RDD number of RDDB2025877443.

### Statistical analysis

Data were analyzed using the GraphPad Prism (Graphad Software), version 8.0. Comparisons between two groups were analyzed using a two-tailed unpaired Student’s *t* test. For multiple-group comparisons, one-way or two-way ANOVA, followed by Tukey’s multiple comparisons test, was used, unless otherwise specified. Tumor growth curves were analyzed by two-way ANOVA, followed by Tukey’s post hoc test. Statistical significance was defined as a *p* value less than 0.05. All statistical analyses were based on at least three independent biological replicates.

## Data availability

All data supporting the findings of this study are included within the article and its supporting information, and additional raw data are available from the corresponding author upon reasonable request.

## Supporting information

This article contains supporting information.

## Conflict of interests

The authors declare that they have no conflicts of interest with the contents of this article.
