# Supplementary material for: The microbial metabolite I3A inhibits ferroptosis and the effectiveness of redox-based cancer therapy
Source: J Biol Chem. 2025 Dec 5;302(1):111004. doi: 10.1016/j.jbc.2025.111004 (PMC12804130; doi:10.1016/j.jbc.2025.111004)
Supplement: Supplementary Material 1 [file mmc1.docx]

**Supplementary Figure 1.** **I3A confers resistance to RSL3-induced ferroptosis and IL4I1 expression correlates with GPX4 in human cancers.**

**A-D.** A375 **(A)**, LS174T **(B)**, ID8 **(C)** and HCT116 **(D)** cells were treated with I3A (500μM), ferrostatin-1 (FER-1,10μM) or RSL3 (1– 6 μM) for 24 h as indicated, then cell viability was detected by CCK-8 assay.

**E-F.** B16 cells were treated with the indicated dose of I3A (50/100/250/500 μM) or RSL3 (1 μM) for 8 h, then cell death was detected by FACS using zombie staining **(E)**, the quantification was shown in **(F)**.

**G.** B16 cells were treated as indicated for 24 h, cell death was detected by LDH assay.

**H-I.** Cells were treated with the indicated dose of I3A or RSL3 (1μM) for 3 h, ROS accumulation was detected by FACS, representative histogram was shown in **(H)**, and mean fluorescence ratio was shown in **(I)**.

**J-L. Correlation between *Il4i1* and *Gpx4* mRNA levels in TCGA cohorts of liver hepatocellular carcinoma (J), skin cutaneous melanoma (K) and colorectal adenocarcinoma (L), normal and tumor samples are plotted separately; Pearson’s r and P values are indicated.**

**M-N. B16 cells were transfected to overexpress IL4I1 or empty vector (EV), the expression of** IL4I1 was detected by WB assay **(M)** and quantitative results were shown in **(N)**.

Data are shown as mean ± SEM. ns denotes not significant; *** *P* < 0.05*;* **** *P* < 0.0001.** Statistical comparisons were performed using one-way or two-way ANOVA analysis followed by Dunnett’s multiple comparisons test. The results are representative of at least three independent biological replicates.

**Supplementary Figure 2. AHR is required for the anti** **- ferroptotic action of I3A.**

**A.** The free radical scavenging capacity of I3A (500 µM), I3P (200 µM) or ferrostatin-1 (FER-1, 10 µM) were detected by DPPH radical‐scavenging assay.

**B.** Volcano plot of RNA-seq data from B16 cells treated with I3A (500 µM) versus vehicle for 3 h; red points highlight validated AHR target genes.

**C-D.** Cells were treated with I3A (500 µM) or RSL3 (1 µM) for 24 h, then cell viability and death was assessed using crystal violet staining assay **(C)** or LDH assay **(D)**.

**E-F.** The knockout and re-expression efficiency of AHR was detected by WB assay **(E)** and quantitative results were shown in **(F)**.

**G-H.** sgScr and sg*Ahr* B16 cells were treated with **I3A** (500 µM)**, I3P** (200 µM), **FER-1** (10 µM) **or RSL3** (1 µM) **for 24 h,** cell viability was assessed using crystal violet staining assay **(G)** and cell death was detected by LDH assay **(H)**.

**I-J.** sgScr and sg*Ahr* B16 cells were treated with **I3A** (500 µM)**, I3P** (200 µM) **or RSL3** (1-6 µM) **for 6h,** cell viability was detected by CCK-8 assay.

Data are shown as mean ± SEM. ns denotes not significant; **** *P* < 0.0001. Statistical analysis was performed using one-way or two-way ANOVA followed by Dunnett’s multiple comparisons test. The results are representative of at least three independent biological replicates.

**Supplementary Figure 3.****I3A suppresses c-Jun induction independently of Hmox1.**
**A-K.** B16 cells were treated with I3A (500 µM) or RSL3 for 3 h, the expression levels of AKT/P-AKT, mTOR, PTEN, HIF1A and P53 were detected by Western blot **(A,D,F,H,J)**, and quantitative results were shown in **(B,C,E,G,I,K)**.

**L-M.** The expression of P53 following siRNA transfection was measured **(L)**, and quantitative results were shown in **(M)**.

**N.** B16 cells were transfected with siP53 or negative control (NC) for 48 h, then treated with I3A (500 µM) or RSL3 (1 µM) for 24 h, cell viability was assessed using crystal violet staining assay.

**O.** B16 cells were treated with LW6 (HIF1A inhibitor, 20 µM), I3A (500 µM) and RSL3 (1 µM) for 24 h, cell viability was assessed using crystal violet staining assay.

**P.** B16 cells were treated with RSL3 (1 µM) or I3A (500 µM) for 2 h, RNA-seq analysis was performed, and the top 15 differentially expressed genes were visualized in a heatmap.

**Q.** WT-B16 or sgAhr cells were co-transfected with an AHR-responsive XRE‐Luciferase reporter and either WT or dE3 AHR plasmids for 24 h, followed by DMSO or I3A (500 μM) treatment for additional 24 h, then XRE‐luciferase activity was measured.

**R.** sgScr and sgHmox1 B16 cells were treated with I3A (500 µM) or RSL3 (1 µM) for 24 h, cell viability was assessed using crystal violet staining assay.

**S.** qPCR validation of *Hmox1* knockdown in sg*Hmox1* versus control sgScr B16 cells (normalized to 18S).

Data are shown as mean ± SEM. ns denotes not significant; * *P* < 0.05; *** *P* < 0.001. Statistical comparisons were performed using one-way followed by Dunnett’s multiple comparisons test. The results are representative of at least three independent biological replicates.

**Supplementary Figure 4.** **RSL3 does not affect the mRNA expression of GPX4.**

B16 cells were treated with RSL3 (1 or 2 µM) for 2 h, then the mRNA expression of Gpx4 was detected by qPCR (normalized to 18S).

Data are shown as mean ± SEM. ns denotes not significant. Statistical comparisons were performed using one-way followed by Dunnett’s multiple comparisons test. The results shown were representative results of at least 3 biological replicates.


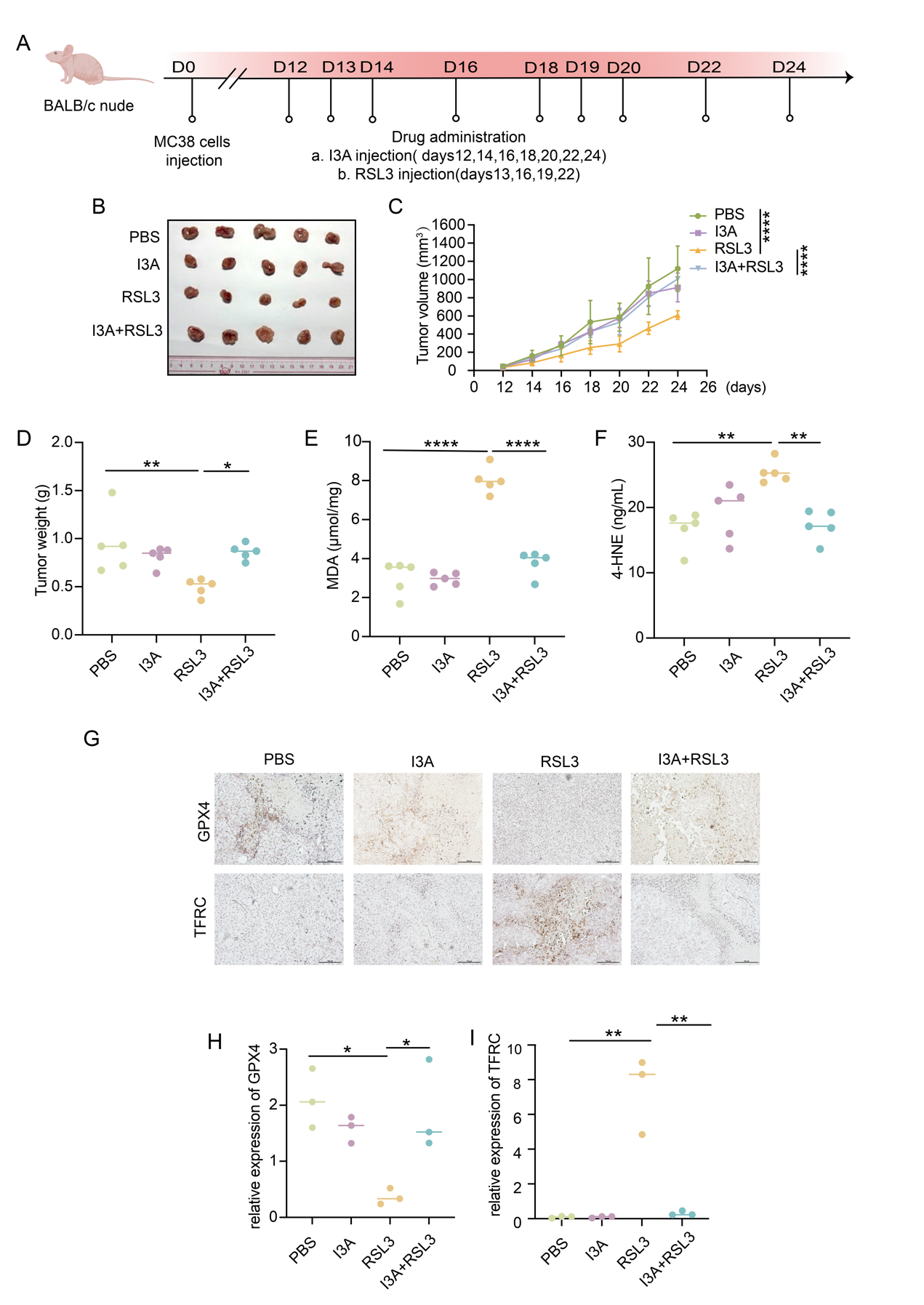


**Supplementary Figure 5. I3A compromises the anti-tumor efficacy of RSL3 in MC38 xenografts.
A.** Schematic illustration of the *in vivo* experimental design on mouse colorectal tumor model; MC38 cells (1x10^6^ per mouse) were subcutaneously inoculated on BALB/c-nude mice (Day 0), followed by intraperitoneal (i.p.) injection of RSL3 (50mg kg^−1^) or vehicle solvent in combination with I3A (50mg kg^−1^) or vehicle solvent on the indicated days, n=5 in each group.

**B-D.** Mice were i.p. injected with I3A/vehicle in combination with RSL/vehicle, tumor growth was monitored every other day, then the tumor images **(B)**, tumor growth curves **(C)**, and final tumor weight **(D)** were shown, the endpoint was defined when the tumor volume reached 2000 mm^3^.

**E-F.** At the endpoint, tumors were isolated, and quantification of lipid peroxidation markers including MDA**(E)** and 4-HNE **(F)** were detected with commercially available kits.

**G-I.** GPX4 and TFRC from tumors were detected by immunohistochemistry (IHC) analysis **(G)**, scale bar = 100 µm, and the quantification of GPX4 and TFRC immunoreactivity (H-score) were shown in **(H, I)**.

Data are shown as mean ± SEM. * *P* < 0.05; ** *P* < 0.01; **** *P* < 0.0001. Statistical analysis was performed using one-way ANOVA followed by Tukey’s multiple comparisons test.

**Supplementary Table 1.**

**Sequences of sgRNAs or siRNAs**

| gene | Primer F | Primer R |
| --- | --- | --- |
| sg*A**hr* | CACCGCGAAATCCTGACCTACGTGC | AAACGCACGTAGGTCAGGATTTCGC |
| sg*Hmox1* | CACCGAGATGGCGTCACTTCGTCAG | AAACCTGACGAAGTGACGCCATCTC |
| si*Ahr*-1 | GCTCAGGAATTTCCCTACAAA | TTTGTAGGGAAATTCCTGAGC |
| si*Ahr*-2 | AGAGCTCTTTCCGGATAATAA | TTATTATCCGGAAAGAGCTCT |
| si*Gpx4-1* | AUGCCAUCAAAUGGAACUUUATT | UAAAGUUCCAUUUGAUGGCAUTT |
| si*Gpx4-2* | GACGUAAACUACACUCAGCUATT | UAGCUGAGUGUAGUUUACGUCTT |
| si*P53-1*  si*P53-2* | CCAGAAGAUAUCCUGCCAUTT  GGAAGUCCUUUGCCCUGAATT | AUGGCAGGAUAUCUUCUGGTT  UUCAGGGCAAAGGACUUCCTT |

**Supplementary Table 2.**

**The qPCR primer sequences**

| gene | Primer F | Primer R |
| --- | --- | --- |
| Gpx4 | CACCGCGAAATCCTGACCTACGTGC | AAACGCACGTAGGTCAGGATTTCGC |
| LC3 | CACCGAGATGGCGTCACTTCGTCAG | AAACCTGACGAAGTGACGCCATCTC |
| Hmox1 | TCAGGCAGAGGGTGATAGAA | GCTCCTGCAACTCCTCAAA |
| 18S | TTCCGATAACGAACGAGACTCT | TGGCTGAACGCCACTTGTC |
| Total bacteria | ACTCCTACGGGAGGCAGCAG | ATTACCGCGGCTGCTGG |
| Lactobacillus reuteri | TTGGAAATGTTCCACAAGAC | TTGTGAGTTTGGATTGAACC |
| 16s | AAACTCAAAKGAATTGACGG | CTCACRRCACGAGCTGAC |

**Supplementary Table 3.**

| gene | Primer |
| --- | --- |
| M-AHR-dE3-F | CTGGCGGCCCCTCAGAGCTCTTTAACAATTCAACTTTGCTGAACTC |
| M-AHR-dE3-R | GAGTTCAGCAAAGTTGAATTGTTAAAGAGCTCTGAGGGGCCGCCAG |

**Supplementary Table 4.**

**Abbreviations**

| Abbreviation | Full definition |
| --- | --- |
| DMSO | Dimethyl sulfoxide |
| TRP | L-Tryptophan |
| L-KYN | L‐Kynurenine |
| KYNA | Kynurenic acid |
| 3-OH KYN | 3‐Hydroxy‐DL‐kynurenine |
| 5-HIAA | 5-Hydroxyindole-3-acetic acid |
| 5-MIAA | 5-Methoxyindole-3-acetic acid |
| I3P | Indole‐3‐pyruvic acid |
| 3-IAA | Indole‐3‐acetic acid |
| IA | Indole lactic acid |
| I3A | Indole‐3‐aldehyde |
| I3C | Indole‐3‐carbinol |
| 3-HAA | 3-Hydroxyanthranilic acid |
| 5-HTP | 5-hydroxytryptophan |
| 5-MeO-DL-Trp | 5-Methoxy-DL-tryptophan |
| N-Ac-Trp | N-Acetyl-L-tryptophan |
| 5-HT | 5-hydroxytryptamine |
| IDA | trans-3-indoleacrylic acid |
| FER-1 | Ferrostatin-1 |
| AHR | Aryl hydrocarbon receptor |
| GPX4 | Glutathione peroxidase 4 |
| IL4I1 | IL4-induced gene 1 |
| WT | Wild type |
| DPPH | 2,2-Diphenyl-1-picrylhydrazyl |
| LDH | Lactate dehydrogenase |
| ROS | Reactive oxygen species |
| MDA | Malondialdehyde |
| 4-HNE | 4-Hydroxynonenal |
| TFRC | transferrin receptor |
